# The Prosthetic Workflow in the Digital Era

**DOI:** 10.1155/2016/9823025

**Published:** 2016-10-18

**Authors:** Lidia Tordiglione, Michele De Franco, Giovanni Bosetti

**Affiliations:** ^1^Department of Surgical and Morphological Science, University of Insubria, 21100 Varese, Italy; ^2^Private Practice, Abbiategrasso, Milan, Italy; ^3^Private Practice, 21100 Varese, Italy

## Abstract

The purpose of this retrospective study was to clinically evaluate the benefits of adopting a full digital workflow for the implementation of fixed prosthetic restorations on natural teeth. To evaluate the effectiveness of these protocols, treatment plans were drawn up for 15 patients requiring rehabilitation of one or more natural teeth. All the dental impressions were taken using a Planmeca PlanScan® (Planmeca OY, Helsinki, Finland) intraoral scanner, which provided digital casts on which the restorations were digitally designed using Exocad® (Exocad GmbH, Germany, 2010) software and fabricated by CAM processing on 5-axis milling machines. A total of 28 single crowns were made from monolithic zirconia, 12 vestibular veneers from lithium disilicate, and 4 three-quarter vestibular veneers with palatal extension. While the restorations were applied, the authors could clinically appreciate the excellent match between the digitally produced prosthetic design and the cemented prostheses, which never required any occlusal or proximal adjustment. Out of all the restorations applied, only one exhibited premature failure and was replaced with no other complications or need for further scanning. From the clinical experience gained using a full digital workflow, the authors can confirm that these work processes enable the fabrication of clinically reliable restorations, with all the benefits that digital methods bring to the dentist, the dental laboratory, and the patient.

## 1. Introduction

It is very frustrating for dentists to complete a prosthetic rehabilitation, whether complex and simple, only to discover that the oral situation does not correspond to the dental technician's casts.

A wide variety of materials and techniques are available for taking impressions of the oral cavity.

The complexity consists in acquiring critical knowledge of the various impression materials (polyethers, polyvinyl siloxanes, polysulfides, hydrocolloids and others) in terms of both their chemical-physical characteristics and their different clinical use and handling [1].

In the age of digital dentistry, we can now replace these various impression materials with an intraoral scanner [2].

Intraoral scanners represent the first step in a totally digital process of design and fabrication of dental prostheses.

The possibility of implementing prosthetic restorations, on both natural teeth and implants, using fully digital workflows is now a reality, and any clinic can avail of the modern digital methods to improve its daily clinical practice.

The advantages of the digital method not only are in terms of the new range of materials that can be used but also can be seen along the entire workflow, from impression and design, for completion of the prosthesis [3–6].

The methods for scanning dental arches using intraoral systems provide comparable impressions to traditional materials in terms of clinical accuracy [7], eliminating operator-dependent variability, reducing the time and treatment costs for rehabilitation, and improving patient compliance [8–11]. There are also several benefits for patients: scan requires less time than taking conventional impressions, the images can be immediately analysed by the operator, and it can be easily repeated, if necessary, either entirely or partially. In addition, the dimensions of the scanner tip make it much more comfortable in terms of encumbrance than the use of traditional materials, particularly for patients with a sensitive gag reflex or fear of choking [12–14].

The purpose of this paper is to present a fully digital workflow based on the latest techniques, now widely validated, from digital scan of intraoral impressions and processing with CAD dental design software to fabrication through the prototyping of diagnostic crown and subtraction milling of the finished prosthetic tooth in order to standardise the workflow and minimise possible errors.

## 2. Materials and Methods

### 2.1. Patient Selection

This is a clinical follow-up examination of 14 patients who had been given single-unit or multiple-unit zirconia and lithium disilicate crowns and veneers by seeking to make the best use of the new working methods and implement fully digital workflows. The clinical phases were conducted in the period from January 2014 to December 2014.

The clinical cases were selected based on the following parameters: eight patients requiring fixed prosthetic rehabilitation on individual natural teeth were initially selected to perfect the technique and reduce the author's learning curve, three more patients were then selected needing prosthetic rehabilitation on two adjacent teeth, followed by two patients requiring work on up to four teeth distributed between both dental arches and, finally, a complicated prosthetic rehabilitation on natural abutments was completed on a patient with multiple abrasions, loss of vertical dimension, and reduction in clinical crown height.

Patient's inclusion criteria were age of at least 18 years, without any systemic pathologies, sufficient oral hygiene, low caries activity with less than five new restorations during the preceding five-year period, with no signs of active bone resorption, furcation involvement, periapical pathology, or mobility. Only natural teeth or fixed prosthesis without treatment needs was accepted as antagonists. Root canal therapy had been conducted, where necessary, on the selected teeth at least six months before the prosthetic rehabilitation, and they had to be symptom-free with no X-rays signs of periapical lesion. Teeth that had still been vital, with no need for root canal therapy, had remained vital. Periodontal health was required: probing depth <4 mm, maximum grade 1 mobility, and no vertical bone pocket.

### 2.2. Intraoral Scan

All the impressions required for the implementation of the arranged treatment plans were taken using a Planmeca PlanScan intraoral scanner (Planmeca OY, Helsinki, Finland). This is a device that relies on short wavelength laser light projection (450 nm). The scanner does not require the application of powder on the intraoral surface and it has several interchangeable autoclavable tips, in various sizes, to suit the clinical situation. It can be used simply by connecting it to a computer or prepared dental unit, allowing it to be operated easily in various workstations. In addition, the scanning software allows digital casts to be exported in the open STL format, giving the clinician complete freedom in the management of the subsequent stages of the prosthetic rehabilitation using Exocad software (Exocad GmbH, Germany, 2010).

To validate the procedure, two steps were taken during the first cases: prototype casts were created in order to test the accuracy of the prosthetic restoration, and resin diagnostic crowns were made to be tested and modified, if necessary, directly in the patient's mouth. After verifying the reliability of the technique, we decided to eliminate these two steps.

### 2.3. The Full Digital Workflow

**Figure 1:**
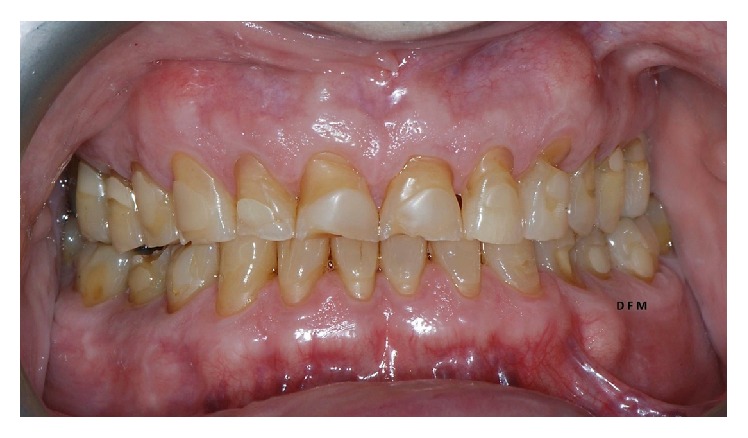
Intraoral preoperative conditions. Notice widespread cervical abrasions and abfractions.

**Figure 2:**
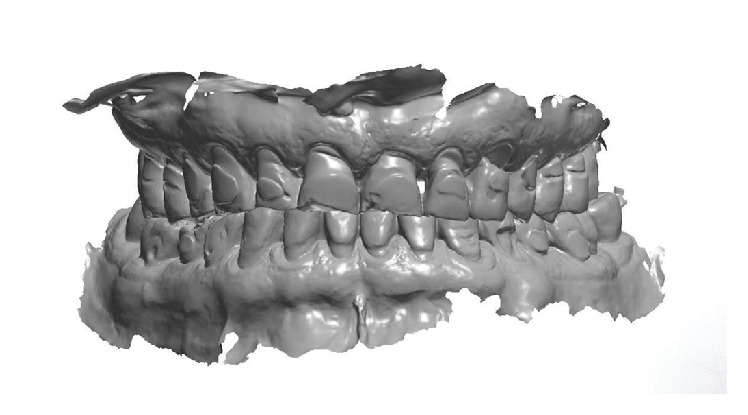
3D image of the preoperative situation originated from the intraoral scan.

**Figure 3:**
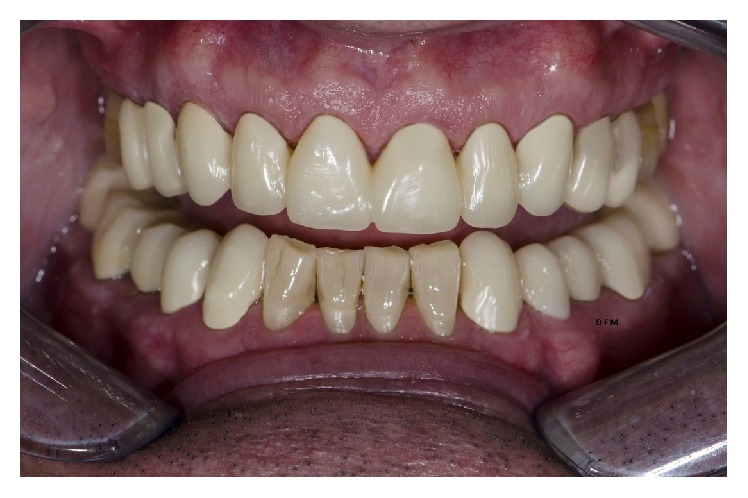
Clinical evaluation of the aesthetic and functional outcome of the provisional.

**Figure 4:**
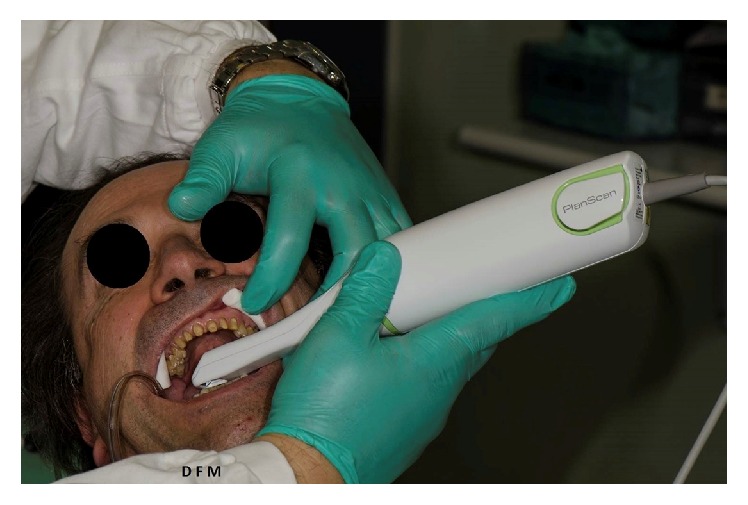
Intraoral digital scan of the preparations of both dental arches.

**Figure 5:**
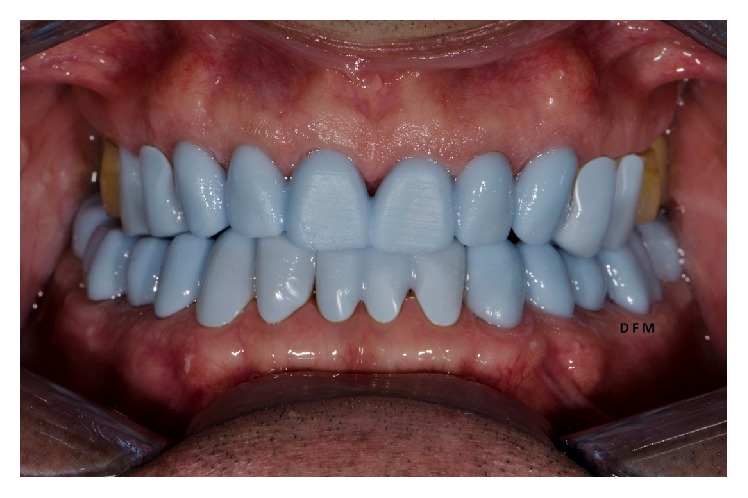
Clinical test and evaluation of the definitive crowns design through the 3D printed crowns: analysis of the finishing lines, interproximal areas, occlusal contacts, and aesthetic result.

**Figure 6:**
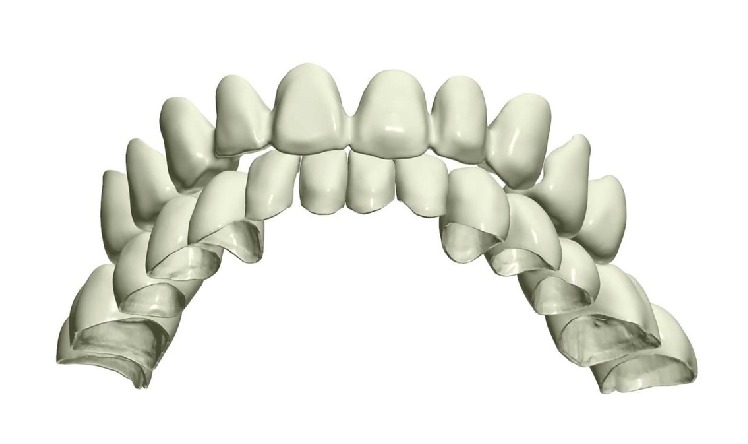
Development of the 3D design project of the definitive crowns.

**Figure 7:**
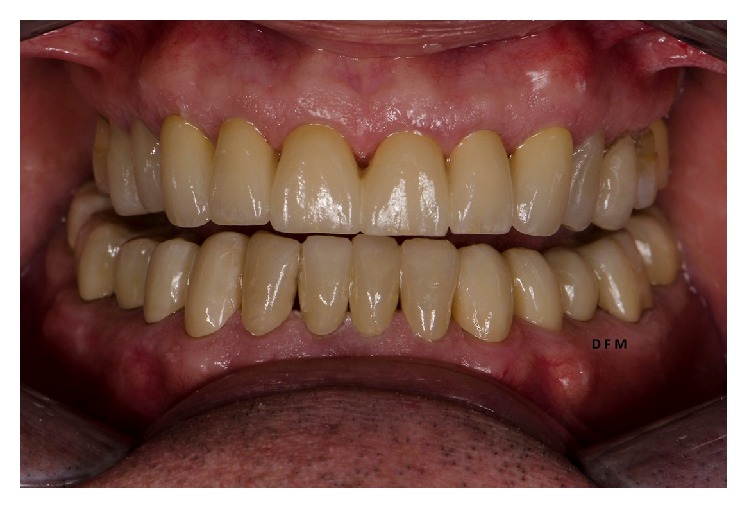
Clinical intraoral result after cementation.

A total of four clinical appointments were initially required:

 (i) Scanning of the arches performed with the intraoral scanner (IS) to allow the design of the temporary crown based on the digital casts (Figures 1 and 2). All the scanning was performed using a Planmeca PlanScan (Planmeca Oy, Helsinki, Finland), which does not require the use of opacifying powder. We first produced a scan of the entire arch involved as far as the second molar, followed by a scan of the opposite arch with a similar extension, and then a scan of both arches in the maximum intercuspation position (MIP), which allowed the imaging software to immediately relate the two scans.
 (ii) Dental preparation and adjustment of the resin temporary crown milled from PMMA are carried out (Figure 3); scanning of the final intraoral impression is performed if the marginal periodontium was not affected by the fitting of the temporary crown; otherwise scanning was scheduled for the following session. The final scanning was similar to that described for the temporary crown (Figure 4). The only difference was that two gingival retraction cords were positioned in the gingival sulcus of the prepared teeth, with the more coronal one removed a few moments before taking the impression.
 Temporary PMMA restorations were cemented with eugenol-free cement (Temp Bond NE, Kerr GmbH, Rastatt, Germany, or Freegenol, GC, Bad Homburg, Germany).
 (iii) Try-in procedure is as follows: fitting the resin (Figure 5) and making any necessary occlusal and/or proximal adjustments on the diagnostic crowns.
 The diagnostic crowns were obtained using the 020D DigitalWax® rapid prototyping machine (DWS, Vicenza 2007), featuring a BluEdge® laser source, which combines high speed with precision and surface quality. It also has a vertical positioning device that allows the base of the modelling platform to emerge to an extent corresponding to the thickness of the solidified layer, thanks to its synchronised laser. The photosensitive resin for DigitalWax RD096 stereolithography systems (DWS, Vicenza 2007) was used, which provides high definition, high resolution, and durability.
 A third scan was then performed and imported into the CAD software Exocad (Exocad GmbH, Germany, 2010) in order to transfer on the digital project any clinical adjustment achieved on the diagnostic crown. This third scan was performed with an intraoral scan of the diagnostic crowns in the right position and an extraoral scan of the crowns themselves focusing on the cervical design.
 (iv) Delivery and cementing of the final prosthesis are carried out (Figures 6 and 7).
 The materials selected for the construction of the prostheses were lithium silicate and monolithic zirconia due to their biocompatibility, biomechanical behaviour, aesthetic qualities, and diamagnetic characteristics.
 All the final prostheses, both monolithic zirconia single crowns and vestibular lithium disilicate and three-quarter lithium disilicate vestibular veneers with palatal extension, were fabricated using a Granite 5-axis numerically controlled milling machine by Dental Machine (Dental machine, Piacenza, Italy). All 5 axes are interpolated continuously and managed by electronically controlled brushless digital motors, with automatic current and position management, and are able to work with all materials (wax, PMMA, various resins, composite material, presintered zirconia or aluminum oxide, lithium disilicate titanium (grades 2 and 5), and Cr-Co alloy).
 All the zirconia monolithic crowns were milled out of Zirite® discs (Kéramo, Tavernerio, Como, Italy): isostatic pressed partly sintered zirconium oxide stabilized with yttrium and colored by Colorodent NANO-ZC® (Orodent, Castelnuovo del Garda, Verona, Italia) colorant immersion for 3–8 seconds depending on the thickness of the restoration and sintered according to the manufacturer's instructions in a Nabertherm sintering furnace (Nabertherm GmbH, Lilienthal, Germany).
 Lithium disilicate veneers were milled from IPS® e.max pressed ceramics blocks (Ivoclar Vivadent, Schaan, Liechtenstein), colored with IPS e.max CAD Crystall Shades (Ivoclar-Vivadent, Schaan, Liechtenstein), and crystallized in ceramic furnaces following the manufacturer's instructions.
 The marginal fit of the abutments was assessed with a silicone pressure indicator paste (Fit Checker, GC, Bad Homburg, Germany).
 The lithium disilicate restoration surfaces were etched with 5% hydrofluoric acid (IPS Ceramic Etching Gel, Ivoclar Vivadent, Schaan, Liechtenstein) for 60 seconds and then rinsed and air-dried. Then silane Monobond Plus (Ivoclar Vivadent) was applied and allowed to react for 20 seconds. The inner restoration surfaces of Zirconium crowns were sandblasted and cleaned in an ultrasonic unit for about 1 minute and then rinsed and air-dried. Abutment surfaces were conditioned with Multilink Primer A/B mixed in a 1 : 1 ratio and applied and scrubbed for at least 30 seconds. All restorations were luted adhesively with a self-curing luting composite Multilink Automix (Ivoclar Vivadent, Schaan, Liechtenstein).
 Finally, static and dynamic occlusions were checked with a 40 *μ* microthin Articulating Papers (Bausch, Köln, Germany).
 Initially, a total of four clinical appointments were required: preparation and adjustment of the temporary crown, scanning for the intraoral impression, testing of the diagnostic crown, and delivery and cementing of the final prosthesis. Later on, when the operators had gained more experience, we were able to avoid the clinical test on the diagnostic crown and reduce the appointments to three by applying occlusal and proximal adjustments directly to the final prosthesis.

### 2.4. The Follow-Up

The clinical examination and radiographic checkups were scheduled for one month after the final cementing, six months, and one year.

The recall examination was accomplished by the two operators that conducted the treatments. Both biological and technical parameters were recorded during each recall.

Biological parameters included probing depths of the abutment teeth, its papilla bleeding index (PBI), secondary caries, endodontic complications, and fractures of the abutment teeth. Technical evaluation included fracture or chipping of the crown or veneer, loss of retention wear or surface roughness, and aesthetic characteristics.

The parameters recorded for vestibular veneers, three-quarter vestibular veneer with palatal extension, and crowns were the same.

## 3. Results

A total of 44 prostheses were installed in 14 patients (8 women and 6 men). Of these, 8 patients were selected for single crown rehabilitation on posterior teeth with milled monolithic zirconia (5 women and 3 men); 5 patients needed multiple unit prosthetic rehabilitation: of these 2 required monolithic zirconia crown on two adjacent posterior teeth (2 men), 1 woman required two lithium disilicate vestibular veneer on anterior teeth, and two women needed zirconia monolithic crown on three teeth distributed between both dental arches. Finally, complete prosthetic rehabilitation was accomplished: it consisted of 10 monolithic zirconia single crowns (8 on posterior teeth and 2 on anterior teeth), 10 lithium disilicate vestibular veneers, and 4 lithium disilicate vestibular veneer with palatal extension (Table 1).

None of the final prostheses made using the digital method needed adjustment of the occlusal and interproximal surfaces, while the occlusal adjustments to the diagnostic crowns were entirely comparable to those performed on prosthetic devices made in the traditional manner.

No failure of the prosthetic component occurred on any of the crowned teeth. In one single case, there was a fracture of a three-quarter vestibular veneer with palatal coverage on one premolar, attributed to an occlusal overload. There was no need to reprocess the digital cast, as the CAD STL processing file was used for remilling, so that the veneer could be directly replaced. After balancing the tooth contact as well as possible and instructing the patient to use a protective night guard, the problem no longer occurred.

## 4. Discussion

CAD/CAM digital processing, just like traditional processing, requires a digital cast from which the restoration can be designed. The digital cast can be obtained by digitising a physical cast, generated from traditional impressions, or based on a cast created digitally using an intraoral scanner.

Both the laboratory scanner and the intraoral scanner provide clinically acceptable accuracy for the creation of prosthetic structures [15, 16]. The former, however, is affected by intrinsic errors and variability due to the use of conventional impression materials and their development in plaster. Intraoral scans can greatly facilitate and standardise dental impression taking also in more difficult cases such as full-arch scans [17] and even in the case of edentulous arches [18], although further clinical studies would need to be conducted for these types of clinical cases. It should be noted, however, that there is some variability with regard to the precision and accuracy of various intraoral scanning systems [19].

The advantages of using the digital method also include considerable saving of time and steps, both for the dentist in the clinic and for the dental technician in the laboratory. Compared with traditional impressions, digitalised impressions dispense with the need to select an impression tray, the consumption of material, the disinfection of the material and the impression tray, and the need for packaging and delivery of the impression. Thanks to this technique, the laboratory can now dispense with the steps of casting the impression and refining the cast and the use of articulators.

A study by Lee and Gallucci has also shown that the digital method is noticeably more appreciated by operators than the traditional method and that its application enables considerable savings in time [20].

Acquisition and mastery of the technique by the operators was much simpler and more efficient, given the possibility of modifying and rescanning during the course of the work [21].

CAD/CAM processing methods were among the first digital innovations to be introduced to dentistry in around 1980 [22]. They allow processing of traditional materials, such as cobalt-chromium alloys, composite and acrylic resins, feldspathic ceramics, some reinforced glass ceramics, and wax through subtraction processes, using CNC milling machines [23]. These machining processes are faster and less expensive compared to traditional processing, while maintaining an excellent level of quality [24]. One of the main benefits of using CAD/CAM methods, however, is the possibility of working with materials that otherwise could not be used in dentistry. These materials include titanium, titanium alloys, and polycrystalline ceramics such as zirconium oxide.

Thanks to its characteristics, zirconium oxide has now become one of the most widely used materials for the fabrication of fixed prostheses with CAD/CAM [25] processing. It has high resistance to bending and fracture, a low specific weight compared to other prosthetic materials, and diamagnetic behaviour in the event that an MRI scan is required. However, its aesthetic superiority to metal structures, the possibility of being more conservative with hard dental tissues, as thicknesses can be reduced compared to those of metal-ceramic crowns, its excellent biocompatibility, and the possibility of achieving metal-free restorations are the reasons for the increase in its use. The biocompatibility of zirconia has been demonstrated in numerous in vitro [26] and in vivo [27, 28] studies, which have shown the absence of cellular genome mutations and other adverse reactions in biological tissues exposed to the samples of zirconium oxide. These same studies show that its high biocompatibility is mainly due to its intrinsic ionic stability, which minimises the inflammatory response. Zirconia is now used for the production of prosthetic substructures, implant abutments, monolithic restorations including inlays, crowns, bridges and full arches, and orthodontic brackets [29].

Contrary to what is often believed, the milling of zirconium oxide requires a very reliable and structurally stabile removal system. In fact, when milling a disk of the material in the green state, the speed, force, and movements of the machine have to be controlled and supervised in order to prevent fractures (visible or invisible and very insidious) and the oversizing of undercuts.

For this reason, we used a 5-axis industrial milling machine with tools capable of ensuring excellent milling precision on thicknesses of 1.5 tenths, thanks to their structural stability and shrink-fit mounting system.

This result, after sintering in the kiln and first coloring to bring out its potential translucency, proves visibly and palpably to be a surprisingly thin artefact, capable of adapting perfectly to the anatomical structure of the patient's tooth.

This study is concordant with the literature already available in affirming that the possibility of digitally designing a prosthetic artefact directly from the scanned model, thus avoiding the traditional steps of taking an impression and casting the model, reduces the possibility of error and improves the marginal precision of the prosthesis and the proximal contacts [30].

## 5. Conclusion

The authors agree with the literature in affirming that the entire digital workflow, from the scanning of the impression to the fabrication of the prosthetic product, has been proven to be reliable and reproducible in clinical practice.

Although further comparative studies on the precision and accuracy of intraoral scanners may be necessary, the clinical outcome, improved patient compliance, and saving of time and materials achieved with this method represent a significant advance in prosthetic dentistry, and the choice of innovative materials that it allows is undoubtedly an important step in scientific and biological progress.

## Figures and Tables

**Table 1:** Number of single zirconia crowns (Zn); multiple unit prosthetic rehabilitation with zirconia crown (Zn) and lithium disilicate vestibular veneer (DSL vest. veneer); complex rehabilitation with single zirconia crown (Zn), lithium disilicate vestibular veneer and lithium disilicate vestibular veneer with palatal extension (DSL 3/4 veneer) and their distribution in posterior (P: premolars, molars) and anterior (A: incisors and canines) areas.

Single crown	Multiple unit		Complex rehabilitation			
Zn	Zn	DSL vest. veneer	Single Zn crown		DSL vest. veneer	DSL 3/4 veneer
P	P	A	P	A	A	P
8	10	2	8	2	10	4
